# The Impact of Postgraduate Health Technology Innovation Training: Outcomes of the Stanford Biodesign Fellowship

**DOI:** 10.1007/s10439-016-1777-1

**Published:** 2016-12-21

**Authors:** James Wall, Eva Hellman, Lyn Denend, Douglas Rait, Ross Venook, Linda Lucian, Dan Azagury, Paul G. Yock, Todd J. Brinton

**Affiliations:** Stanford Byers Center for Biodesign, Stanford University, 318 Campus Drive, E100, Stanford, CA 94305 USA

**Keywords:** Innovation, Biomedical engineering, Biomedical technology, Health technology, Innovation Fellowship, Biodesign

## Abstract

Stanford Biodesign launched its Innovation Fellowship in 2001 as a first-of-its kind postgraduate training experience for teaching biomedical technology innovators a need-driven process for developing medical technologies and delivering them to patients. Since then, many design-oriented educational programs have been initiated, yet the impact of this type of training remains poorly understood. This study measures the career focus, leadership trajectory, and productivity of 114 Biodesign Innovation Fellowship alumni based on survey data and public career information. It also compares alumni on certain publicly available metrics to finalists interviewed but not selected. Overall, 60% of alumni are employed in health technology in contrast to 35% of finalists interviewed but not selected. On leadership, 72% of alumni hold managerial or higher positions compared to 48% of the finalist group. A total of 67% of alumni reported that the fellowship had been “extremely beneficial” on their careers. As a measure of technology translation, more than 440,000 patients have been reached with technologies developed directly out of the Biodesign Innovation Fellowship, with another 1,000,000+ aided by solutions initiated by alumni after their training. This study suggests a positive impact of the fellowship program on the career focus, leadership, and productivity of its alumni.

## Introduction

The Program in Biodesign was launched in 2001 at Stanford University with a mission to help train the next generation of leaders in biomedical technology innovation. Its first educational offering was the Biodesign Innovation Fellowship—a 10.5 month, full-time training experience designed to teach aspiring biomedical technology innovators a rigorous, need-driven process for developing new medical devices and preparing to deliver them into patient care. Over 15 years, Stanford Biodesign’s definition of biomedical technology innovation has expanded beyond traditional medical devices to include device-based diagnostics, digital health solutions, and health information technologies. In parallel, its educational offerings have grown to include a portfolio of graduate and undergraduate courses, an executive education program, international fellowships, a fellowship for Stanford faculty, and a program to train faculty members from universities around the world. All of these initiatives focus on teaching a stepwise, need-driven approach developed at Stanford, called the biodesign innovation process.^1^,^7^ At its core, this process involves three key phases: (1) identifying and screening important unmet healthcare needs; (2) inventing and vetting new technologies to address them; and (3) developing detailed implementation plans to bring the products to market. Some Stanford Biodesign courses provide training on the end-to-end process, while others tackle specific aspects in more depth (e.g., regulatory and clinical trials, healthcare economics, and global considerations).

The cornerstone of the overall program remains the Biodesign Innovation Fellowship. Each year, fellows are selected through a competitive screening process from applicants with experience in medicine, engineering, and/or business functions ranging from design to marketing to operations. These candidates are postgraduates: engineers typically have completed a Master’s or PhD degree, physicians have usually finished their residency or a medical fellowship, and some candidates have multiple degrees. Of the 100+ applicants that Stanford Biodesign screens annually, approximately 24 finalists are invited for two-days of intensive in-person interviews. From this group, 12 individuals are selected for the program based on their education, evidence of creativity or inventiveness in their training or early careers, and perceived ability to contribute to a multidisciplinary team. Teams of four fellows each are then assembled with the goal of mixing backgrounds and skill sets. The scope of the fellowship has increased over time to its current size of three teams per year.

Each academic year, a different clinical area is chosen as the focus, and faculty and community clinicians in that field are recruited to support the training and mentoring of the fellows.^4^,^6^ The training program begins with a “boot camp,” during which the fellows concentrate on three primary areas of learning: (1) lectures from experts in the chosen clinical area; (2) didactic instruction on the fundamental activities in the biodesign innovation process that span needs finding through concept screening; (3) execution of a “mini-project” to gain first-hand experience in applying what they are learning about these activities. Following boot camp, the fellows repeat the fundamental steps in the biodesign innovation process on projects in the chosen clinical area for the remainder of the fellowship year. They begin with one month of immersion in clinical practice to identify unmet healthcare needs based on direct observations. Typically, each team generates more than 200 needs during this period. These needs are then researched and screened through an approach that focuses on numerous variables including market size, patient impact, pathophysiology, competitive landscape, and the opportunity to create value for healthcare stakeholders. Ultimately, needs with the greatest potential for innovation rise to the top of the list for further investigation. At this point, a full 3–4 months into the training experience, the fellows begin brainstorming solutions. They generate multiple concepts for each of their top needs using a variety of ideation techniques. Next, they screen these concepts against factors such as technical feasibility, intellectual property, regulatory and reimbursement pathways, and potential business models to identify and evaluate key risks. The concepts with the best comparative risk profile are taken forward into development and implementation planning. This portion of the fellowship depends heavily on mentorship from real-world health technology innovators and executives to help improve the translational potential of the projects and ensure that the learning experience is practical.

Beyond Stanford, numerous multi-disciplinary programs have been developed in the US and worldwide using similar needs-driven design approaches to health technology innovation.^2^ While in some cases these programs have published the descriptions of technologies invented by the trainees, no rigorous study has been undertaken to understand the impact of health technology innovation training on *careers* of the alumni.^3^,^5^ Stanford Biodesign described the outcomes of its program in terms of start-up companies founded, funds raised, jobs created, and patients reached in an interim report in this journal.^1^ However, that work did not address the impact of the program on the subsequent careers of its trainees. This new study examines the career focus, leadership, and productivity of Stanford Biodesign alumni, as well as qualitative perceptions of the program’s effectiveness. As a part of this analysis, career metrics of the Biodesign Innovation Fellowship alumni were compared to those of finalists who ultimately were not selected for the program. While this is clearly an imperfect comparison in important respects, the intent was to explore any large differences in the career trajectories of these two groups that might lead to a better understanding of candidate selection and/or impacts of the training program.

## Methods

This study was a retrospective review performed under a Stanford Institutional Review Board (IRB) waiver that determined that the survey of fellowship alumni did not meet federal definitions of research or clinical investigation. Data were collected from three sources: (1) publically available career information, (2) a survey of Biodesign Innovation Fellowship alumni, and (3) a survey of Biodesign trainees who have launched companies out of the program. Data from all three sources were gathered for alumni of the Biodesign Innovation Fellowship who completed the program between June 2002 and June 2015 (*n* = 114). Additionally, publically available career information was collected for finalists (*n* = 120) who interviewed for the Stanford fellowship but were not selected to participate in the program for the years in which the names of these finalists were available. Importantly, the individuals performing the data collection from public sources could not be blinded to the differences between alumni and finalists because of their familiarity with at least some of the names of the fellowship alumni. Additional data collection details for each source are outlined below.

### Career Information from Public Data Sources

Current job titles and company names were collected from public sources for the entire cohort of Biodesign Innovation Fellows to determine the types of career paths that alumni have taken. Similar data were collected from public sources for finalists who interviewed for the Biodesign Innovation Fellowship but ultimately were not selected for the program in the application year or any subsequent application years (the names of these candidates were available only for the academic years ending in 2005 through 2008, and 2010 through 2015). LinkedIn (http://www.linkedin.com) was the most commonly used source. Other web searches were used to gather supplemental information (e.g., from biographies on company or university websites). Specifically for alumni, when no public data were available for an individual, a personal email was sent to determine their current role and company. Additionally, gender and academic degrees completed before the fellowship were collected from public sources and checked against original fellowship application materials when available. An alumni representative from each fellowship year then re-examined collected data on fellowship alumni for accuracy.

Based on their primary role, each subject was assigned into different categories for career type, functional role, and type of leadership. For career type, individuals were considered to be working in health technology if their role was related to medical devices, device-based diagnostics, digital health solutions, and/or health information technology. Job titles were used to assign each alumnus to a functional role (e.g., general management, R&D/engineering/design, clinical/regulatory/quality, marketing/sales, business development, operations/finance, continued academic training, academic faculty, clinical practice, or other). General management included top-level positions that did not fit within a specific functional area including CEOs, principals, and presidents. Job titles were also used to assign individuals into leadership categories (e.g., executive officer, vice president, director, manager, or individual contributor). In certain cases, assumptions were made to standardize across position titles (e.g., a principal at a small consulting firm was put in the executive officer category). The individual contributor category included individuals whose primary job title did not fit into any other leadership category (e.g., staff engineer, consultant). Organizations where individuals are employed were similarly placed into categories based on size of organization, type of organization, and type of health technology (if applicable). Type of health technology was only assigned for smaller organizations with one or a few products. The categories included devices, device-based diagnostics, and digital health. Health information technology solutions were considered part of the digital health category.

### Fellowship Alumni Online Survey

In an effort to capture information that is not available through public sources, the authors developed a 33-question online survey to administer to alumni of the Biodesign Innovation Fellowship. The instrument was tested for face validity by a group of selected fellowship alumni. In the survey, health technology was defined the same as in the public data collection approach. Leadership was self-determined in one of three categories for an individual’s current primary role: (1) leadership position with full-time direct reports; (2) leadership position without full-time direct reports, or (3) individual contributor.

For questions regarding companies founded by Biodesign Innovation Fellowship alumni, qualifying entities were required to have raised at least $250,000 in external grant and/or private capital funding, secured their intellectual property from Stanford or other relevant owner, and completed incorporation. For companies founded based on work initiated after the completion of the fellowship (rather than based on work initiated during their training), alumni were asked to quantify total funding raised and patients reached to date. These data were collected in ranges, and the lowest value in each range was used for calculating a total across all alumni.

Alumni were also asked a series of qualitative questions, including their opinions about how beneficial the fellowship program had been on their careers, how well it achieved specific educational goals, how effective it was in teaching select skills, and the extent to which it had positively affected their career trajectories. For all such questions, a scale of 1–5 was used with 5 being the highest response and 1 being the lowest.

Three emails were sent between February and March 2016 to all Biodesign Innovation Fellowship alumni requesting their participation in the survey. The survey was created in and disseminated through the Qualtrics platform (Qualtrics, Provo, UT).

### Targeted Survey of Company Founders

An additional survey of 12 questions was administered in March 2016 to Biodesign trainees (including Biodesign Innovation Fellowship alumni) who founded companies based on work initiated during their training with Stanford Biodesign. The same qualifying requirements for these companies were used as in the larger fellowship alumni survey. With regard to funds raised and patients reached, these trainees were asked to provide a specific total for each company (rather than a range) and these values were summed to determine the collective total. Participants for this survey were identified based on prior knowledge, as well as results from the fellowship alumni survey. This company tracking survey was created in and disseminated through the Qualtrics platform (Qualtrics, Provo, UT).

Two-tailed *z*-tests comparing population proportions were used to determine a statistical difference. A *p* value of less than 0.05 was considered significant.

## Results

Public career data were compiled for all 114 Biodesign Innovation Fellowship alumni graduating the program between 2002 and 2015 (100%) and for all 120 finalists who interviewed for the fellowship but were not selected for the program from the years 2005–2008 and 2010–2015, which were the years for which finalist names were available. The survey of fellowship alumni was answered by 95% of fellowship alumni (*n* = 108). The targeted survey of company founders was answered by 100% of relevant individuals (*n* = 41).

Individuals completing the Biodesign Innovation Fellowship have diverse educational backgrounds. They include 44 MDs representing more than 20 specialties, 63 engineers (BS, MS, PhD) across a variety of disciplines, and 7 others with graduate degrees in business or public health and/or doctoral degrees in the sciences. Collectively, 27 of these individuals have had multiple degrees of relevance (e.g., MD/Engineering, MD/MBA, Engineering/MBA). The educational backgrounds between alumni and finalists for the fellowship were notably different, with more clinicians accepted into the program and more PhDs interviewed but not accepted (Table 1).

**Table 1:** Educational background and gender at the time of application for alumni and finalists interviewed but not selected for the program.

	Alumni (*n* = 93) (%)	Finalists (*n* = 120) (%)
Highest degree obtained		
BS	8	2
MS	25	21
MBA	5	9
MD	38	19
PhD	25	47
Unknown	0	3
Gender		
Female	23	27

### Career Focus

As determined from public data, a total of 94% (*n* = 107) of all Biodesign Innovation Fellowship alumni (*n* = 114) work in either clinical medicine or health technology, with another 4% (*n* = 5) in biotech or pharma. Of the 32% (*n* = 37) in clinical medicine, 65% (*n* = 24) work as faculty members or are in continued training at an academic medical center.

**Figure 1:**
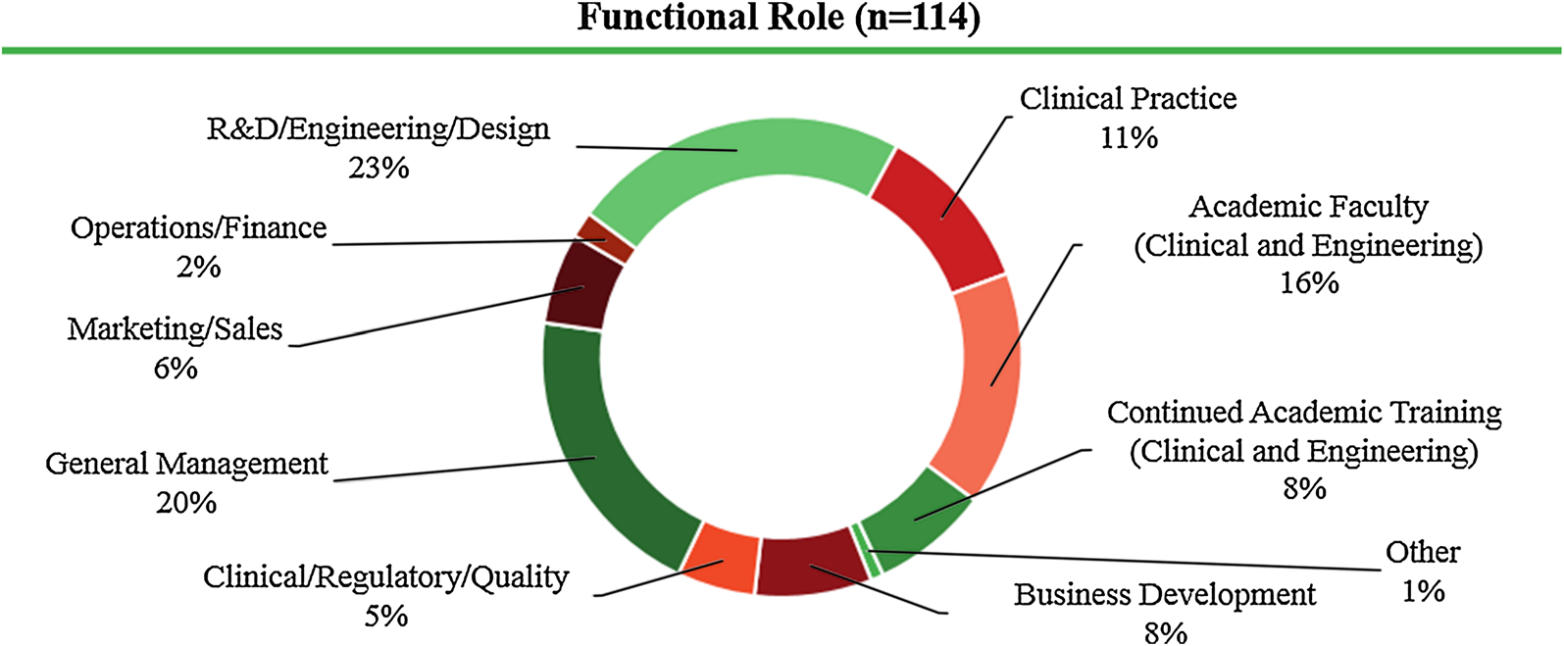
Current functional roles of Biodesign Innovation Fellowship alumni.

Fellowship alumni hold diverse functional roles in general management, R&D/engineering/design, clinical/regulatory/quality, marketing/sales, business development, operations/finance, continued academic training, academic faculty, clinical practice, and other (Fig. 1).

Further evaluation of just those Biodesign Innovation Fellowship alumni actively working within the health technology sector (61%, *n* = 70) reveals that 80% (*n* = 56) work at health technology companies; the rest work in health technology inside a variety of organization types, including advisory firms (incubators, consulting, and law) (9%, *n* = 6), biotechnology or technology companies with health technology divisions (4%, *n* = 3), universities with health technology-related training programs (4%, *n* = 3), or other (3%, *n* = 2).

For alumni working specifically within health technology companies (49%, *n* = 56), 45% of this subgroup (*n* = 25) are employed by start-ups with less than 10 employees. The rest are in established health technology organizations as follows: 34% small (11–100 employees, *n* = 19); 4% medium (101–1000 employees, *n* = 2), and 18% large (>1001 employees, *n* = 10). Of the start-ups and small companies (that tend to have a single product/platform focus, *n* = 44), 68% (*n* = 30) are in medical devices, 27% (*n* = 12) in digital health, and 5% (*n* = 2) in device-based diagnostics.

**Figure 2:**
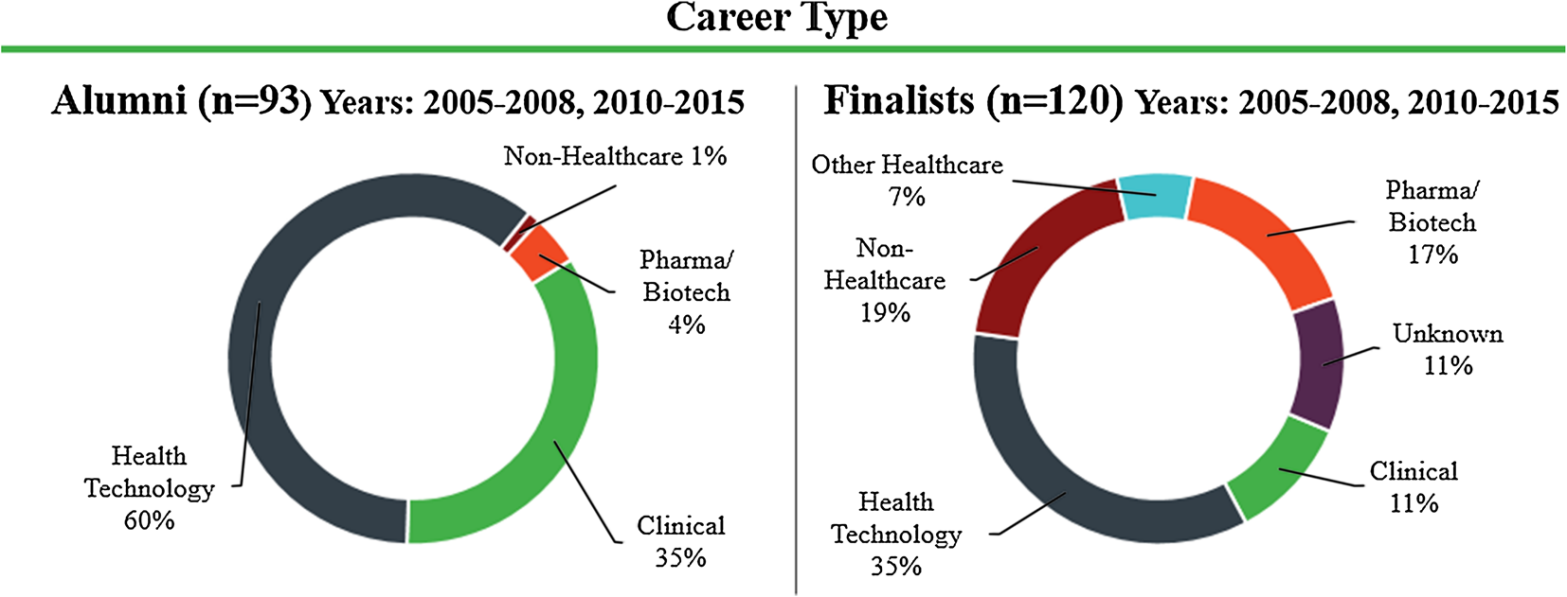
Comparison of career type matched by year for Biodesign Innovation Fellowship alumni (left) and finalists (right) interviewed but not selected for the fellowship.

Specifically for the subset of fellowship alumni (*n* = 93) who could be matched in the same years with finalists interviewed but not selected for the program (*n* = 120), 60% (*n* = 56) are currently employed in the health technology field compared to 35% (*n* = 42) of finalists. Only 1% (*n* = 1) of this alumni subset works outside healthcare in contrast to 19% (*n* = 23) of the finalists (Fig. 2).

### Leadership

A total of 69% (*n* = 74) of all Biodesign Innovation Fellowship alumni responding to the online survey (*n* = 108) self-reported that they were currently in leadership roles, and 49% (*n* = 53) indicated that they had full-time direct reports, with an average of 6.2 direct reports per manager. When divided into three cohorts by graduation year to understand if leadership roles increased over time, alumni show significant growth in leadership positions as their careers progress; 59% (*n* = 23/39) of alumni in the most recent cohort hold leadership positions as compared to 68% (*n* = 30/44) and 84% (*n* = 21/25) in the older two groups. There was a statistical difference in leadership roles between the 2002–2006 cohort and the 2012–2015 cohort (*p* = 0.035).

**Figure 3:**
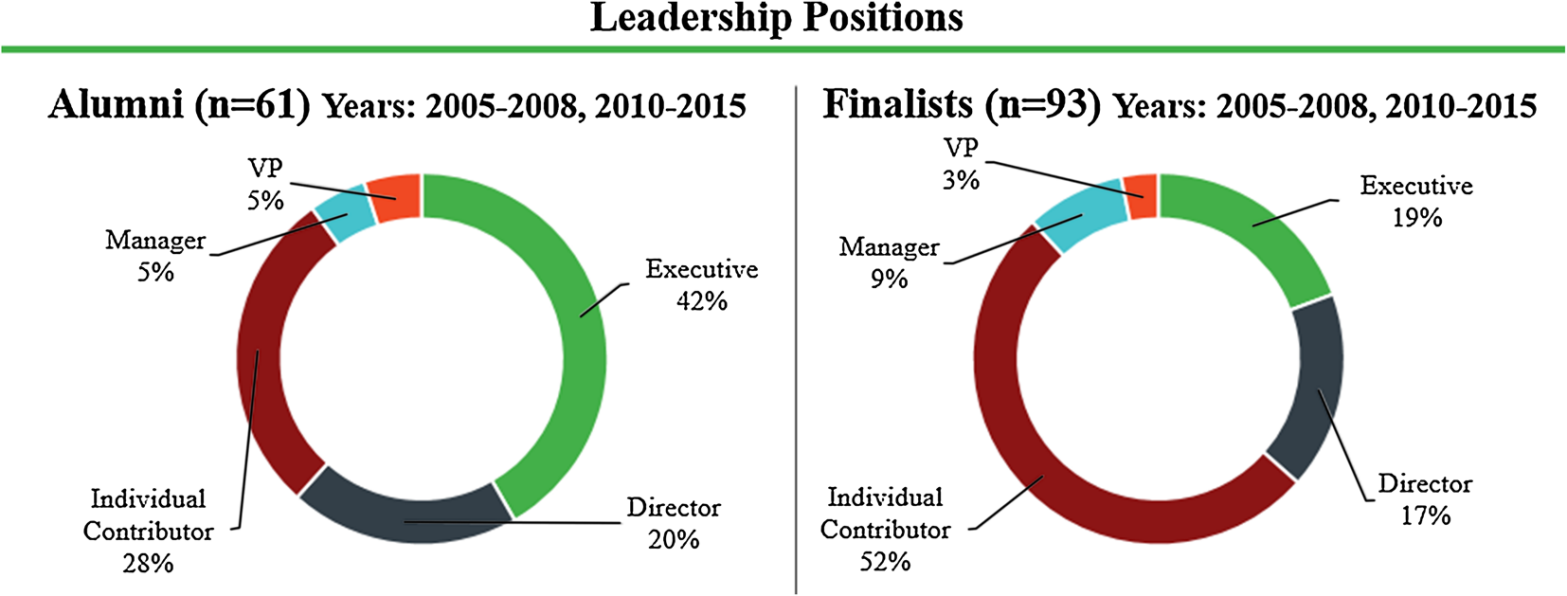
Leadership positions as determined by job title for Biodesign Innovation Fellowship alumni and finalists interviewed but not selected for the program, excluding clinicians and those in unknown roles.

In order to assess the leadership positions of alumni fellows vs. finalists who were not selected for the fellowship, position titles for non-clinicians were compared between the alumni and finalist groups for the years when records of the finalist names were available. Among alumni (*n* = 61), 28% (*n* = 17) were individual contributors and 72% (*n* = 44) were managers and above. In the control group of finalists (*n* = 93), 52% (*n* = 48) were individual contributors and 48% (*n* = 45) were managers and above (Fig. 3). There was a statistical difference between the alumni and finalist groups (*p* = 0.003).

In an effort to assess the consistency of the leadership information gathered from public sources vs. the survey, we compared data from these two sources for the alumni group (for which both sets of data were available). In the public data approach, which did not capture leadership roles for clinicians, 70% (*n* = 54/77) of alumni were identified as managers and above. Comparatively, in the survey, 61% (*n* = 44/72) of alumni working in health technology self-identified as managers, and 85% (*n* = 61/72) identified themselves as leaders in their roles (with or without direct reports). Taking into account the range of the self-reported metrics, the survey results and the data from public sources are directionally in alignment for these alumni, with some small potential for the overestimation of management positions using the public data approach.

### Productivity

At the time of the analysis, a total of 41 companies were formed from the combined Biodesign educational programs, of which 61% (*n* = 25) were founded from the Biodesign Innovation Fellowship (the remainder were from the Stanford Biodesign global fellowships and graduate student classes). At the time the targeted survey of company founders was completed, the companies launched from the fellowship were responsible for 460 new jobs in health technology and for helping 440,739 patients. Beyond the fellowship, many alumni have gone on to repeat entrepreneurial activity in health technology, which has led to the creation of 273 additional jobs and 1,031,100 more patients helped (Table 2). This self-reported data cannot be independently verified, but the authors believe that it represents a reasonable estimation based on their knowledge of the relevant companies.

**Table 2:** Companies founded by Biodesign trainees.

Type of company	Number of companies	Number of fulltime employees	Funding raised ($)	Patients reached
Companies founded from the Biodesign Innovation Fellowship	25	460	$278,833,559	440,739
Companies founded by Biodesign Innovation Fellowship alumni based on work after graduation	33	273	$260,416,000	1,031,100

**Figure 4:**
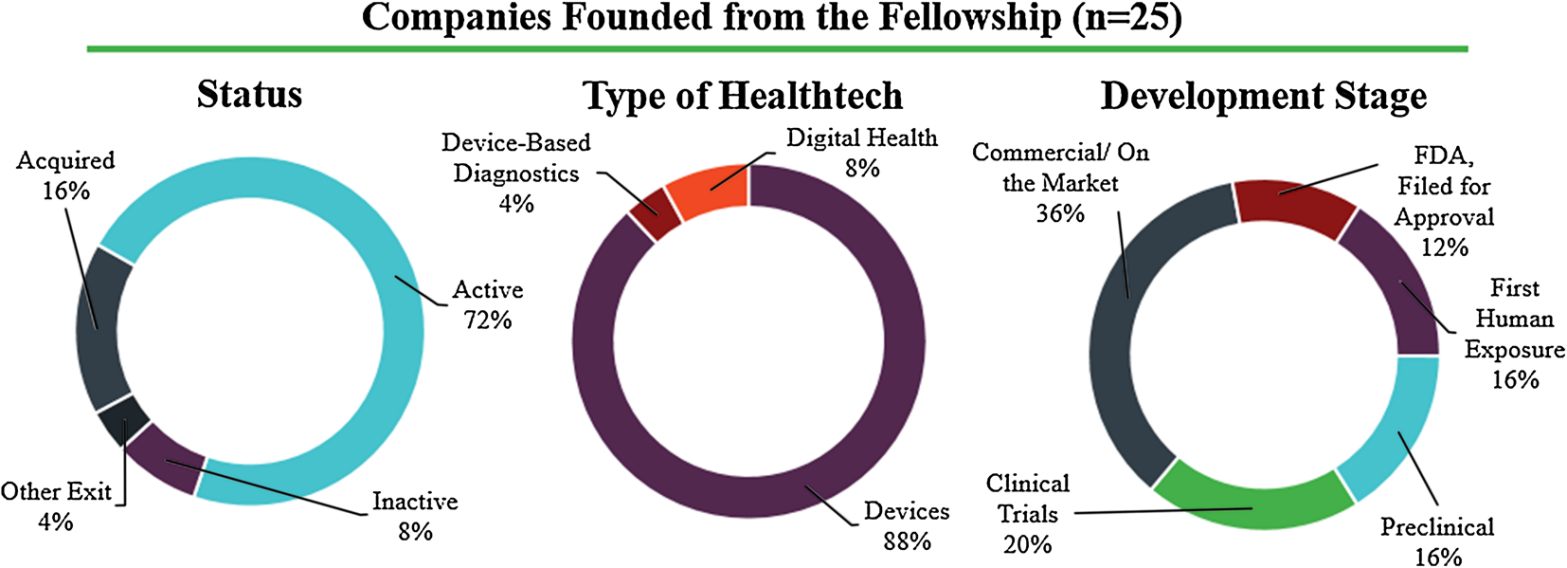
Current status, area of focus within health technology, and stage of development for companies founded out of the Biodesign Innovation Fellowship.

Additional data were gathered regarding the current status of the companies formed directly out of the fellowship. Among these companies, 72% (*n* = 18) remain active and an additional 20% (*n* = 5) have been acquired or achieved another business exit. A total of 88% (*n* = 22) of the companies are in the medical devices space, 8% (*n* = 2) are in digital health, and 4% (*n* = 1) are in device-based diagnostics. Among the active companies, 36% (*n* = 9) are currently fully on the market, another 12% (*n* = 3) are pursuing FDA filings, 16% (*n* = 4) are in early human use, and 36% (*n* = 9) are preclinical or in clinical trials (Fig. 4).

Another career metric addressed in the study is the involvement of alumni in teaching or training activities. Overall, 24% (*n* = 27/114) of all alumni primarily work or train in academia. Beyond that, 82% (*n* = 89/108) of those completing the survey self-reported that they have led Biodesign-related educational activities that resulted in a combined total of more than 6000 training experiences (Table 3). (Note that “training experiences” do not directly equate to a total number of people trained since trainees may have participated in more than one training encounter.) Each fellowship alumnus engaged in training (*n* = 89) has educated an average of 76 individuals. In the category of other advisory responsibilities, 75% (*n* = 81) of fellows have advised health technology companies, either by acting as a consultant or subject matter expert, clinical investigator, and/or member of a board of directors.

**Table 3:** Percentage of fellowship alumni who have led different types of training related to the biodesign innovation process and the number of training experiences they have provided.

	Formal training			Informal training	
	Workshop 1 day in duration	Multi-day workshop	Multi-week or multi-month course	Fewer than 10 meetings	More than 10 meetings
Fellowship alumni trainers	30%	17%	22%	72%	39%
Total training experiences	2210	1448	889	1408	830

With respect to publications, a total of 41% (*n* = 44) of alumni are listed as authors on peer-reviewed articles related to health technology innovation for a total of 341 authorships. In the realm of intellectual property, 72% (*n* = 78) of alumni are authors on issued health technology patents for a total of 866 citations.

### Effectiveness of Program

**Figure 5:**
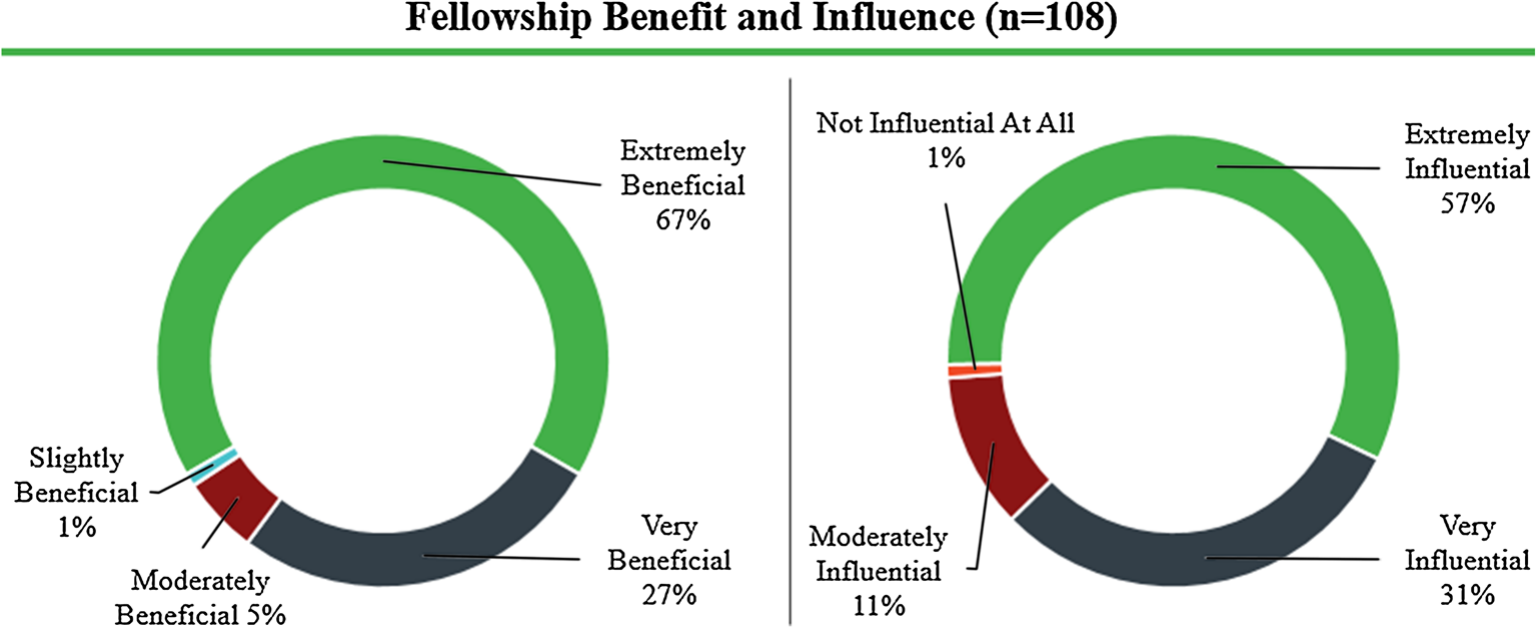
Self-reported benefit (left) and influence (right) of the Biodesign Innovation Fellowship on alumni careers.

All alumni participating in the survey (*n* = 108) responded that the fellowship had benefited their career, with 67% (*n* = 72) stating that it was “extremely beneficial.” In a related question regarding the role of the program in influencing career direction, 99% (*n* = 107) reported some influence, and 57% (*n* = 62) indicated that it was “extremely influential” (Fig. 5).

With respect to the program’s success in achieving specific training goals, the percentages of fellows reporting that it was “very” or “extremely” successful were as follows: (1) teaching a repeatable, disciplined process for addressing unmet clinical needs (94%, *n* = 101), (2) providing access to valuable network of advisors and mentors (96%, *n* = 104), (3) preparing the fellows to work effectively on multidisciplinary teams (90%, *n* = 97), and (4) preparing the fellows for leadership roles in health technology innovation (76%, *n* = 82). In terms of skills acquired as a result of the fellowship, the percentages of alumni who reported that the program was “very” or “extremely” successful were: (1) need finding, research, and filtering (94%, *n* = 102), (2) design thinking and related ideation techniques (82%, *n* = 89), (3) communicating ideas effectively to a target audience (79%, *n* = 85), (4) understanding, evaluating, and prioritizing risks (78%, *n* = 84), (5) strategic and business planning (68%, *n* = 73), and (6) prototyping and engineering (46%, *n* = 50). Alumni also self-reported the frequency with which they have used each skill set post-fellowship, with the percentage of alumni relying on them daily or weekly as follows: (1) need finding, research, and filtering (34%, *n* = 37), (2) design thinking and related ideation techniques (64%, *n* = 69), (3) communicating ideas effectively to a target audience (66%, *n* = 71), (4) understanding, evaluating, and prioritizing risks (77%, *n* = 83), (5) strategic and business planning (62%, *n* = 67), and (6) prototyping and engineering (36%, *n* = 39).

## Discussion

Although a number of multidisciplinary, design-oriented educational programs in biomedical technology innovation have been launched in universities in the US and abroad,^2^ the impact of this type of training remains difficult to quantify.^3^,^5^ The present study reports the results of a detailed survey of 114 alumni of the Stanford Biodesign Innovation Fellowship, who at the time of the study were between one and 14 years into their careers. The data indicate that the fellowship alumni have been productive in inventing new technologies that have translated into patient care; they have progressed into leadership roles in health technology; they self-report that they value the training they received in the Biodesign approach; and they are actively involved in mentoring others in the this approach. This study also compares the career metrics of the alumni with candidates who were finalists in applying to the program but were not selected to be fellows. Many more alumni than finalists continue to work in the healthcare field generally, although this could be due, at least in part, to a much higher percentage of individuals with MDs in the alumni group than in the finalist group. The more striking difference is the higher proportion of fellowship alumni who continue in careers in health technology innovation compared to the finalist pool. This suggests (but, of course, does not prove) that there is a positive impact of the program on career trajectory, which aligns with the report of the alumni that their training in Biodesign had a major impact on their choice of career.

There are several important limitations to this study. First, the comparison of the careers of individuals who completed the fellowship to finalists who were interviewed but not selected has a clear asymmetry that could only be addressed formally by randomizing the fellowship selection process. Second, the accuracy of the data collected through publically available sources could not be independently verified. This is particularly true for the finalists interviewed but not selected for the fellowship because Stanford Biodesign does not maintain first-hand knowledge of these individuals beyond the application period. Unfortunately, the program also collected limited demographic information about these applicants during the application process. Finally, with regard to information collected* via* survey, there are limitations intrinsic to these self-reported data, including the fact that they, too, cannot be independently verified. For example, the claim that alumni have created new companies that, in aggregate, have reached over 1 million patients is based on self-reporting and cannot be independently confirmed (patient numbers are treated as confidential by the large majority of these start-up organizations).

Overall, it is difficult to determine whether an educational program such as the Biodesign Innovation Fellowship makes a significant difference in the career trajectories and contributions of its trainees. One could reasonably ask if these individuals would excel independent of the training program. Although this study does not definitively answer this question, it suggests that the training program is seen as having a positive influence by its alumni. The Biodesign alumni hold more leadership roles in health technology than the comparison group of talented individuals with similar educational backgrounds. This difference is especially pronounced at the executive level, perhaps due to the high rate of start-up activity among Biodesign Innovation Fellowship alumni. The fellowship alumni unanimously report that the training was beneficial to their careers and a high percentage believe it was influential on their career pathway.

The current study also confirms findings from a prior report^1^ that the Biodesign Innovation Fellows are effective in inventing technologies during their training. However, a key objective of the fellowship is to teach a repeatable process for health technology innovation that alumni can continue to apply in their careers. The fact that more companies have been started (and more patients reached) by fellowship alumni based on work *after* their time at Stanford Biodesign compared to *during* their time in the fellowship suggests that the fellows are applying what they have learned beyond the scope of the program.

The survey data reflecting the alumni’s view of the skills training they received during the fellowship was particularly instructive. The alumni especially valued their training in needs finding, research, and filtering, although they reported that they were not using these skills regularly in their current positions. The fact that the training in the category of prototyping and engineering was clearly ranked weakest among the skills developed provides an important opportunity for improvement in the program.

Reviewing data from the alumni demonstrates additional areas where attention is required moving forward. Most striking of these is that the, the fellowship has been largely male-dominated. The program has made some progress in this respect recently, but is actively reviewing its recruitment and communications strategies to achieve a more equal gender balance going forward. Clearly, another area that calls for significant ongoing effort is refining the methods of assessing the impact of the fellowship training, potentially including prospective methods for more detailed tracking of data from alumni and the comparison group.

While more work needs to be done to truly understand the effects of the Stanford Biodesign Innovation Fellowship, this study suggests a positive impact of the fellowship program on the career focus, leadership, and productivity of its alumni.
